# Cortisol Monitoring Devices toward Implementation for Clinically Relevant Biosensing In Vivo

**DOI:** 10.3390/molecules28052353

**Published:** 2023-03-03

**Authors:** Pavel A. Kusov, Yuri V. Kotelevtsev, Vladimir P. Drachev

**Affiliations:** 1 Center for Engineering Physics, Skolkovo Institute of Science and Technology, 121205 Moscow, Russia; 2 Vladimir Zelman Center for Neurobiology and Brain Rehabilitation, Skolkovo Institute of Science and Technology, 121205 Moscow, Russia

**Keywords:** cortisol continuous monitoring, in vivo biosensor, neuroendocrinology, nanomaterials

## Abstract

Cortisol is a steroid hormone that regulates energy metabolism, stress reactions, and immune response. Cortisol is produced in the kidneys’ adrenal cortex. Its levels in the circulatory system are regulated by the neuroendocrine system with a negative feedback loop of the hypothalamic–pituitary–adrenal axis (HPA-axis) following circadian rhythm. Conditions associated with HPA-axis disruption cause deteriorative effects on human life quality in numerous ways. Psychiatric, cardiovascular, and metabolic disorders as well as a variety of inflammatory processes accompanying age-related, orphan, and many other conditions are associated with altered cortisol secretion rates and inadequate responses. Laboratory measurements of cortisol are well-developed and based mainly on the enzyme linked immunosorbent assay (ELISA). There is a great demand for a continuous real-time cortisol sensor that is yet to be developed. Recent advances in approaches that will eventually culminate in such sensors have been summarized in several reviews. This review compares different platforms for direct cortisol measurements in biological fluids. The ways to achieve continuous cortisol measurements are discussed. A cortisol monitoring device will be essential for personified pharmacological correction of the HPA-axis toward normal cortisol levels through a 24-h cycle.

## 1. Introduction

Cortisol monitoring devices, corticometers, designed for the long-term measurements of cortisol concentration fluctuations in vivo, in interstitial fluid, and blood plasma are in high demand for drug development and the pharmacological correction of the HPA-axis [1,2]. However, the creation of a biosensor matching all of the requirements of biomedical science to such instruments is challenging enough. Cortisol complex pharmacokinetics, its chemical and biological properties, require an analytical strategy aimed at avoiding all the error sources that affect cortisol concentration monitoring in biofluids in a time-resolved manner [3].

Laboratory measurement of cortisol concentrations in the physiological range of tens to hundreds of nanomol/L—in human blood, saliva, and urine—can be achieved by immunofluorescent and other commercially available techniques including ELISA and mass spectroscopy [4]. However, this requires bench-based equipment and trained technicians to perform, and the samples are taken and analyzed in a discrete manner, disturbing the subjected patient and introducing more error.

**Figure 1:**
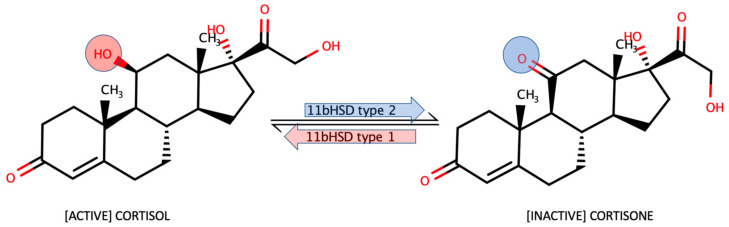
Cortisol–cortisone 11βHSD isoenzyme 1–2 “shuttle”.

A number of techniques have been created and applied to measure cortisol with various sampling techniques in sweat, saliva, tears, urine, however, none of them are efficient enough to be clinically significant or practical enough to be presented on the market and adapted to monitoring and diagnostic devices [5,6,7]. Additionally, the tissue levels of cortisol are affected by cortisol to cortisone interconversion by 11β hydroxysteroid dehydrogenase (11βHSD) enzymes (Figure 1). 11βHSD type 2 is responsible for the conversion of cortisol to inactive cortisone in mineralocorticoid sensitive tissues, while type 1 regenerates cortisol from cortisone in the liver, brain, and some other tissues [8]. The activity of cortisol-converting enzymes in salivary and sweat glands are known to be sources of error, which can be identified by comparing the immunofluorescent assay results with the mass-spectroscopic ones [9].

The activity of the 11βHSD enzymatic shuttle system is currently a target for the development of isozyme-specific inhibitors, preventing inadequate overactivation of the stress response mechanisms under conditions associated with high local cortisol levels such as intracranial hypertension [10,11]. Cortisol molecules are lipophilic and overcome the blood–brain barrier. This steroid exists in blood serum in a dynamic equilibrium of its biologically active free form and biologically inactive portion, selectively reversibly bound to transcortin (corticosteroid binding globulin—CBG) and non-specifically to serum albumins (due to albumin vast quantity compared to CBG concentration in blood). Currently, the quantitative analysis of cortisol in the blood and serum samples in vitro are affected by the facts of the CBG-bound and free cortisol dynamic equilibrium in the sample, temperature, and CBG expression rates in tissues. Some of these issues are currently being addressed by several recalculation techniques—Coolens, Södergård, Dorin, and Nguen equations. However, Molenaar et al. demonstrated the low clinical significance of such measurements and the recalculation results of critically ill patients as well as non-critically ill patients [12]. Authors have commented on such findings by pointing out the fact of the affinity change of CBG due to the NE cleavage of the reactive loop and genetic variations of the CBG gene, decreasing the CBG protein affinity to cortisol.

CBG has one binding site for cortisol with a high (nanomolar) affinity to cortisol (Kd of 33 × 10^−9^ M), being found on the cellular membranes in renal tissue, liver, and others. Some proportion of the total cortisol (up to 5%) is bound to blood albumins non-specifically [13]. The CBG saturation concentration of cortisol is reported to be at the peak level of cortisol at approximately 400 nM/L [14]. The free cortisol portion (up to 5% of total) is biologically active and its concentration fluctuates with certain frequency due to the adrenals’ pulsatory release of cortisol and the CBG–cortisol dynamic depo buffers the influx of hormones into the circulation and target tissues [15]. CBG is possibly involved in the membrane transport of steroids as it was found in the cellular cytoplasm. The spatiotemporal biodistribution of free cortisol is regulated by the local activity of specific proteases—neutrophil elastases during acute trauma, inflammation, or sepsis. CBG expression rates are also important features and regulators of the cortisol complex biodistribution [16,17].

Thus, cortisol is locally enzymatically inactivated and reactivated in sweat and saliva, making the data obtained by non-invasive sampling less accurate and deceiving, while blood puncture itself is a stressful event triggering cortisol release and waking during the sleep hours. Exocrine gland (sweat, saliva, tears—cortisol sample sources) channels are natural dialysis systems excreting only the free cortisol component of the total cortisol as blood carriers are cut off. Due to this, the proportion of cortisol concentration in saliva to that of a serum is different. As a result, biosensors aimed at excreted sample sources will require calibration with parallel measurements of cortisol in the blood or the interstitial fluid (ISF) samples.

High variation can be found in cortisol concentrations measured in the same samples using immunoassays and mass-spectrometry. The hormone concentration is best measured in the specific vascular bed or at the site of its action. This stimulates the development of an implantable cortisol biosensor [18,19]. A biosensor structurally consists of a biorecognition element, which upon binding the analyte molecule, initiates a physico-chemical signal registered by an acquisition element and is transmitted to the receiver. The signal calibration adjustment results in a graphical or digital representation of the analyte concentration in the sensing volume. High interest and demand for the implantable spatiotemporal probe of free (active) cortisol concentrations yielded a plethora of analytical systems potentially applicable for the stated goals.

Nevertheless, there is no medical-grade analytical device present to be used as a continuous cortisol monitoring device (CCM) to support normal hormonal allostasis and prevent acute cortisol-related conditions [20]. The existing continuous glucose monitoring (CGM) devices and their clinical significance are reviewed in the Section 2.1. It serves as an example for the development of the potential CCM device prototype.

## 2. Continuous Monitoring Device

### 2.1. Example of the Clinically Relevant, FDA-Approved Long-Term Implanted Concenration Monitoring Medical Device: Continuous Glucose Monitoring (CGM) Eversense

**Figure 2:**
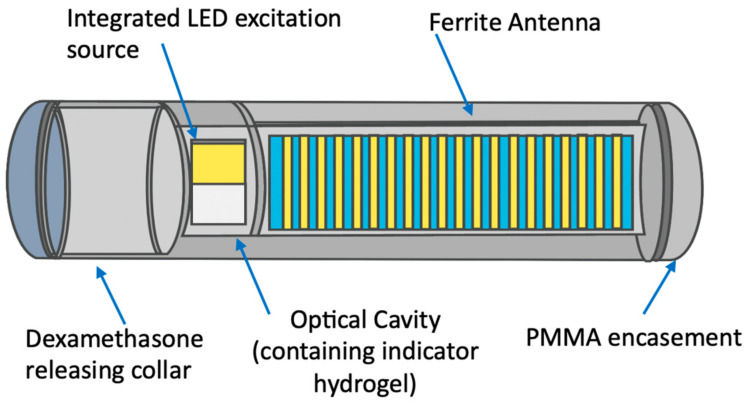
Scheme of the Senseonics Eversense implanted module probe containing one or two (depending on the model—for 90 or 180 days) silicon collars slowly releasing dexamethasone to diminish the inflammatory response in the implantation site. The indicator polymer is in proximity of the optical system that flashes the UV-LED to measure the fluorescence intensity of the indicator gel. The controller performs the measurements and connects to the externally worn transmitter wirelessly to collect and store data points. From [22] with permission from Wiley.

The Seneonics Eversense implanted glucometer consists of an implanted, for up to 180 days, sensing probe connected wirelessly with a matchbox size electronic transmitter, patched to the skin by tape just above the site of the injected (under the skin) 2 cm length polymer capsule (see Figure 2). This is a successful example of such an analytical system and has been proven to be highly efficient in accurate insulin replacement therapy [21].

**Figure 3:**
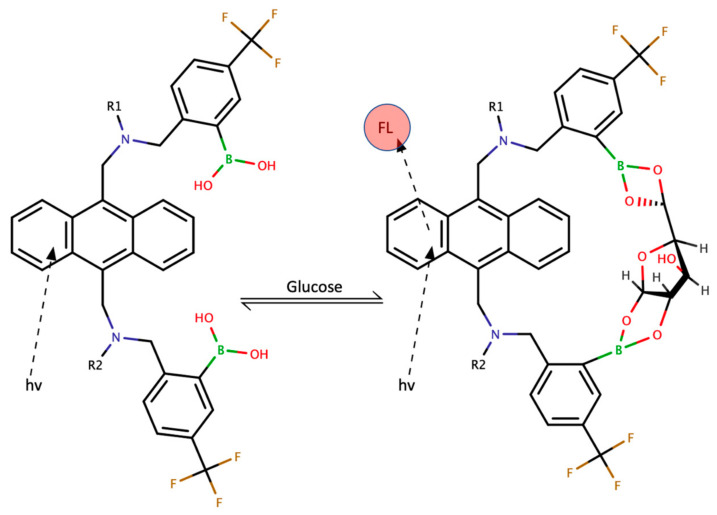
Equilibrium binding of glucose to the indicating polymer. R1 and R2 represent the polymer chain terminals’ “upstream” and “downstream” depicted point. From [22] with permission from Wiley.

The sensor probe is a PMMA capsule containing an analytical system equipped with a medical grade silicone tube secreting anti-inflammatory medicine to protect the capsule from the immune reaction. A small-molecule permeable cap as a semi-membrane allows glucose to passively diffuse to ensure its contact with the sensitive polymer. The indicator component of the gel—polycyclic aromatic molecules—specifically and reversibly bind glucose molecules with a structural reconfiguration allowing the fluorescent signal to be generated by the glucose–indicator complex, as shown in Figure 3. Thus, the fluorescence intensity is proportional to the glucose concentration. Indicator molecules are, in fact, side-branches of the polymer immobilized on the surface of the device sensing area [23].

The capsule is surgically implanted in a patient’s shoulder, belly, or back with a special injection needle by trained medical personnel. A transmitter module is wearable by patching it to the skin over the implanted capsule to perform daily data collection that is represented by a smart watch and smartphone application via Bluetooth. The transmitter reads data from the implanted probe, automatically calibrates and analyzes it, and then stores the data, sending it to the external devices to be read eventually by the user. Further analysis of the long-term monitoring data is performed by a physician to correct the medicine administration regimen and dosage. It was evidentially demonstrated that the usage of a continuous implantable glucose monitor is safe and beneficial to the efficiency of the insulin replacement therapy as well as to the overall functional improvement in the patients’ quality of life [24].

Eversense’s elegant decision to utilize the biorecognition element simultaneously as a signal generation molecule is realized through the equilibrium binding of glucose to the indicator group. Reversibility, selectivity, and fast enough kinetics of the reaction are the keys to the creation of a successful operating time-resolved analytical system. With the first generation CGM Eversense (90 days of safe implantation period), users were required to recalibrate the device each day twice with a finger puncture for blood sampling. The second generation does not longer require daily recalibrations and has two units of inflammation suppressing dexamethasone collars on the implanted probe capsule. It has an extended safe implantation period of up to 180 days, which demonstrates the high practical value and usability of implantable CGM devices to the end user.

### 2.2. Demands of the Continuous Cortisol Monitoring Device in Clinics, Point of Care, and Drug Development

Improvement in the quality of life of people with neuroendocrine conditions through daily monitoring of a biomarker molecule is achievable with a biosensor, as has been used successfully for insulin replacement therapy. An in vivo biosensing analytical system (performing as required by the biological aspects of the task parameters) could also be developed to assess the reactive hypothalamo–pituitary–adrenal (HPA-axis) system of corticoid function regulation with an implantable biosensor for continuous cortisol monitoring (CCM).

There is still no example of a functional, practical, clinically significant, FDA-approved, or under ongoing preclinical study of such an implanted corticometer. However, there is very active research in this field with a number of publications reviewing this topic, annually multiplying the number of articles on novel cortisol biosensor designs, and discussing the future directions on continuous biosensing development [25].

Conditions associated with HPA-axis disruption are disbalancing the normal circadian cortisol secretion pattern and responses with a number of diminishing life quality physiological effects that affect the central nervous system, synaptic plasticity, cognition, and memory [26,27,28].

Chronic inflammation associated with tissue degenerative processes of disease and aging such as arthritis, multiple sclerosis (MS), atherosclerosis, asthma and often genetic disorders (for instance, Duchenne muscular dystrophy (DMD)) are treated with systemic long-term synthetic corticosteroid medicines like prednisone, deflazacort, and dexamethasone [29]. Such treatment could dramatically influence the HPA-axis, causing Cushing-like adverse effects—fatigue, bone fragility, metabolic, circulatory, immune, and nervous systems disruptions [30].

Such patients may benefit from clinically relevant devices aimed to diminish the adverse effects of pharmacotherapy and prevent critical conditions at the point of care. A continuous cortisol monitoring device will help the therapist to analyze the long-term collected data of the users’ HPA-axis response on the change in drug type, combinations, regimens, and dosages [31,32,33]. The goal of such monitoring and therapy is to set the circadian and ultradian rhythms by means of chronotherapy to integrate endogenous cortisol rhythms.

At the moment, pharmacological corrections are prescribed based on less accurate biofluid sampling, dialysis system connection, and technically highly precise mass spectroscopy and tandem mass spectroscopy-chromatography (LS-MS). Such methods tether the patient to heavy, sophisticated laboratory equipment for 24 h or more for monitoring. It may introduce additional errors through the discrete sampling, blood puncture, which disturbs the patient, releasing additional cortisol, and disrupting patients’ sleep at night.

Currently, cortisol daily profiling is performed in diagnostic laboratories as a point-by-point collection of the serum samples for conventional immunoassays or mass-spectrometry methods to measure the free cortisol concentration in vitro. Blood puncture for each-hour determination of cortisol, with further LS-MS measurements or ELISA, requires sampling invasions, influencing the studied patient neuroendocrine responses. Other sample sources such as tears and sweat are less disturbing as they require no sampling procedures. However, these techniques are not practical enough since the patches have no reliable adhesion to the skin. Cortisol exists in urine in free (unconjugated) and conjugated (e.g., glucuronide-conjugated and sulfate-conjugated) forms. Approximately 1–2% of protein-unbound circulating cortisol is excreted in the urine. Therefore, the level of cortisol in the urine to some point reflects the level of the protein-unbound (biologically active or free) form of cortisol in the plasma. Cortisol-converting enzyme activity in the sweat, tears, and saliva make the results of such measurements less reliable. Thus, the most accurate sample sources are blood, serum, or interstitial fluid.

## 3. Perspective Cortisol Biosensors Developed towards Continuous Monitoring: Recognition Elements, Physico-Chemical Phenomena for Signal Generation and Analytical Strategies

### 3.1. Electrochemical Cortisol Sensing

Electrochemical methods include various signal generation techniques based on the measurement of standard parameters of the circuit—potentiometry, amperometry, impedimetry, and voltammetry.

**Figure 4:**
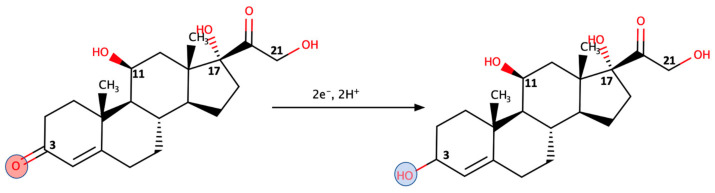
Cortisol reduction is preferable at the C3 carbonyl position of cortisol. Reproduced from [35] under CC BY-NC 3.0.

Non-enzymatic electrochemical cortisol assays can be an interesting direction for CCM biosensor development with the same element functioning as the recognition and the signal generation component. The assay was built on the recent findings of the steroid C3 keto-group being prone to reduction in a preferable manner if its carbonyl is in conjugation with a double bond [34] (Figure 4).

In [34], by formulating a highly efficient reducing substrate of a ZnO layer on graphene oxide, the electrode was created to be sensitive to cortisol in the nanomolar range of concentrations. The described sensor performed in vitro and operated with real saliva with only a 5% error in comparison to the artificial saliva samples calibration. While, this technique was not further applied toward measurements in the blood or ISF, it gives an interesting example of an electrochemical CCM biosensor since no enzyme or specific biorecognition element is used. However, known organic substances selectively reducing cortisol at C3 are not efficient enough in terms of the reaction yield, so further research and optimization could be beneficial for the development of more selective electrochemical sensors for cortisol [35].

Most cortisol biosensors are built on redox or electrochemical changes in the microenvironment of the probe’s surface modified with biorecognition elements. Some strategies have been applied to amplify and transmit a binding event such as resistance change through additional components including structure-switching aptamers, enzymes, and competitive immunoassays with a secondary reporting tracer [36].

Another interesting strategy exists for the electrochemical biosensing of various analytes including cortisol with a biocatalytic reaction of electrode-linked enzyme (oxidases or dehydrogenases are used: as the enzyme catalyzes its reaction, the coenzymes required (NAD+, NADP+, NADH, NADPH, ATP FAD, FADH) are enzymatically modified to be detected and measured by amperometry [37].

The most important obstacle in the way of electrochemical cortisol biosensors in the development of in vivo applications is the sensitivity of the developed systems to the pH and redox potential of the sample matrix. This issue could be solved with sample preparation, prior measurements by filtration, or the introduction of complex membranes selective enough to protect the sensor from the environment, yet still permeable for the free diffusion of amphiphilic cortisol molecules with physiologically relevant rates.

Recent publications have demonstrated an active process of the search for an efficient protective membrane for lipophilic analytes such as steroid hormones [38]. Here, lipophilization of the track membrane (with low selectivity) was performed for both sensor protection from fouling by the biological matrix of the sample and from the pH and redox factors of the medium.

### 3.2. Immunosensors for CCM

A surface-specific time-resolved plasmon-enhanced fluoroimmunoassay (PEF) was developed for cortisol concentration changes over time measurements that aimed to demonstrate the applicability of the novel analytical signal generation technique for the continuous cortisol monitoring task [39]. The system has a limit of detection (LOD) of 0.02 μg/mL and responsivity of about 30 min (the reaction time decreases down to 20 min when performed at 36 °C—normal body temperature). The system can be further transferred to the optical fiber terminal side surface and covered with a polymer capsule with a porous selective membrane to be implanted for in vivo tests in CCM device development. The system’s LOD and responsivity is sufficient to cover the normal cortisol levels in blood, serum, and saliva [3]. The application of the PEF was proposed to be formulated on the surface of the optically active modified surface regions of terminally decladded optical fibers (sensing region) connected through a signal transmission region (the rest of the fiber with intact clad) of the optical fiber connected to the spectrometer and computer through optical connectors. Such back-collecting fiber probe spectroscopy is under development for the CCM task with the already functioning in vitro PEF time-resolved assay. This technique could be a practical, relatively simple, and low-cost to produce sensitive CCM device that is able to read the cortisol fluctuations in the tissue of interest. Note that the proposed probe has a miniature size with a diameter up to 500 µm and length less than 1 cm.

**Figure 5:**
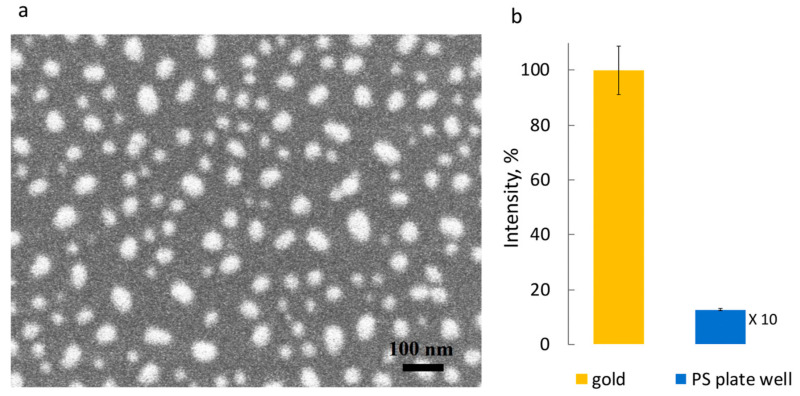
(**a**) SEM image of the plasmon-active surface—Au nanostructured discontinious film on the SiO_2_ chip surface (the task of the time-resolved cortisol monitoring relies on the plasmon enhanced fluorescence phenomenon, which is dependent on the distance between the fluorescence reporter and the surface in the range of tens of nanometers). (**b**) Here, the Au-modified surface demonstrated the ability to generate an analytical signal through 100-fold enhancement of the red (λ_ex_ = 633 nm, λ_em_ = 655 nm) fluorescent reporter intensity in the surface immunocomplex compared to the same immunocomplex fluorescence intensity on the polystyrene well plate (from [39] under CC BY-NC-ND 4.0).

The analytical system consists of the monoclonal antibodies to cortisol, raised against the 3-HS-BSA immunogen with nM affinity to free cortisol. The antibodies are bound to the thin plasmon-supporting film consisting of Au nanostructures on the SiO_2_ chip surface deposited by physical vapor deposition processes (Figure 5).

**Figure 6:**
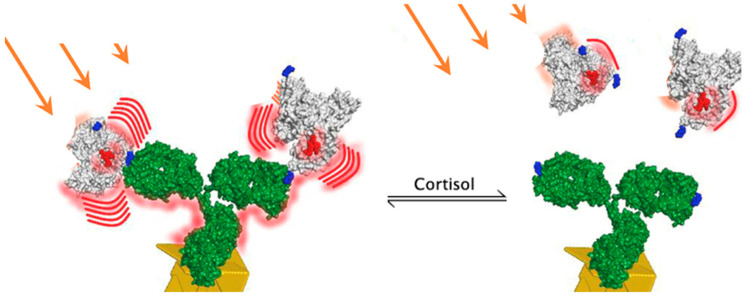
Competitive equilibrium binding of cortisol to the surface-bound monoclonal antibodies to cortisol. Fluorescence intensity of the cortisol–BSA–fluorophore reporter is enhanced in close proximity to the Au nanostructured film surface. When free cortisol is present, the fluorescence intensity gradually drops, allowing the concentration fluctuations to be monitored over time in vitro (from [39] under CC BY-NC-ND 4.0).

The introduction of a competitive displacement assay scheme with surface-bound antibodies to cortisol and bulk mobile reporter element—bovine serum albumin (BSA)—conjugated covalently with cortisol hapten (cortisol-3-HS or cortisol-3-CMO) and fluorophore molecules (FBC) is proposed. Such a scheme allows for recording, with a lag of about 20 min, of the reversible equilibrium binding of the FBC competing with free cortisol for the surface-bound antibody binding sites as the FBC fluorescence intensity is maximal when no free cortisol is present. The FBCs are bound to the antibodies and in close proximity to the Au nanostructured surface. The fluorescence intensity of the system is inversely proportional to the free cortisol concentration in the system. The device can measure the free cortisol concentration variations in the system as FBC is displaced out of the antibody binding sites by free cortisol molecules and passively diffuse out of a ~15 nm (antibody linear size) distance from the Au particles, leaving a surface enhancing area (Figure 6).

The other example of a cortisol immunosensor, and simultaneously cortisol aptasensor, is based on the interaction of surface-bound quantum dots with immobilized antibodies or aptamers that bind cortisol. Upon binding, fluorescence quenching is detected—with an intensity proportional to the bound cortisol concentration—that allows one to measure the cortisol concentration in saliva [40]. Such an immunoassay, being sensitive enough to measure the saliva cortisol concentration changes (whose range in nM/L is less than that in blood or serum—0–14 nM/L), could be further applied to the task of real-time cortisol concentration measurements if further developed. However, the demonstrated assay aims for a less clinically relevant but still practical enough salivary cortisol “one at a time” point measurement.

Another cortisol immunosensor is based on the novel surface substrate, a partially decladded polymer optical fiber (POF) covered with a continuous Au thin film for SPR generation, which demonstrates the applicability of a novel surface and signal transmitting element (optical fiber) for cortisol monitoring. However, the method requires a fiber to be connected to the spectrometer and a light source from both ends, but the sensitive area is suitable for use in vitro for low-volume microfluidic measurements as it is not quite practical to use such fibers to develop implantable devices for in vivo monitoring.

POF mechanical properties are beneficial for in vivo application since its core material—PMMA—makes the hair-thin waveguides flexible and biologically inert as well as prone to covalent modification with various nanochemistries to functionalize its surface. However, even a flexible material such as this is not durable enough to be strained in a loop toward the in vivo measurements by SPR; the fiber loses its waveguide properties and mechanically degrades when sharp angles are introduced to the fiber by bending in such a loop. The spectra of the optical assays were adapted to be used on the tip and side surface of the terminals of the fibers, attracting attention for implantable device development with polymer optic fiber technology [41,42].

**Figure 7:**
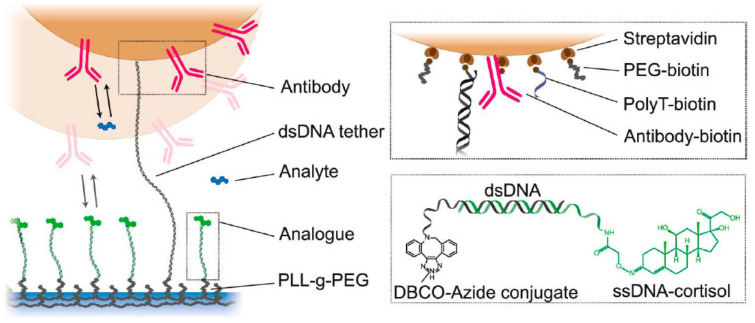
Competitive equilibrium binding of the free cortisol and cortisol hapten bound with a DNA linker to the polymer on the surface. Here, the cortisol concentration influences the mobility of the microparticles bound to the surface—when cortisol is abscent, microparticles are bound tight to the surface, and free cortisol displaces the surface-bound by the short DNA linker cortisol hapten, which could be detected by the analysis of the mobility of the microparticles by optical methods [43]. Reprinted with permission of ACS (https://pubs.acs.org/doi/10.1021/acssensors.2c01358).

The first original study describing the continuous monitoring of cortisol in samples obtained by the microdialysis of blood plasma was recently published [43]. The registration of plasma cortisol interaction with the antibody receptor is achieved by the biosensing of particle mobility (BPM) affected by single-molecule binding and unbinding events [44]. Polymer particles (1 µm in diameter) were functionalized with monoclonal antibodies to cortisol (Abcam G53) This antibody was selected from several commercially available monoclonal antibodies to balance high affinity with the reversibility of binding to the antigen. The particles decorated with G53 antibodies were tethered by long dsDNA to the substrate covered with cortisol-analogues attached to the surface through a short dsDNA linker (Figure 7).

The density of the G53 antibody on the particles was optimized to allow for interaction through a single specific molecular contact between the antibody molecule attached to the particle and a cortisol analogue attached to the substrate. Reversible antigen antibody interaction resulted in binding and unbinding events between the particles, still tethered to the substrate by the long dsDNA molecule. The frequency of antibody mediated binding events was reduced when cortisol was present in the solution. Bound and unbound states of the particles were detected by recording the Brownian movements of 2000 individual particles in the field view of a contrast microscope. It was shown that the sensor responds to cortisol in the high nanomolar to low micromolar range and can monitor physiological plasma cortisol concentrations over 6 h. Displacement curves obtained by increasing and decreasing free cortisol concentrations almost coincided with an IC50 close to 1 μM. This allowed for measurements of the 10 nM increments of cortisol concentration within 10 min intervals, which is suitable for the continuous measurement of physiological fluctuations of the hormone concentration in the plasma. This experiment proved the feasibility of using antibodies as a primary receptor for continuous measurement of the physiological cortisol concentrations. The main limitation of this method is the necessity of the dialysis of plasma as plasma proteins will apparently increase the nonspecific adhesion of the particles to the substrate. Stability of the antibody, particularly of the dsDNA linkers in the biological environment, may also cause a problem. Additionally, it seems to be difficult to adapt the BPM method based on contrast microscopic registration to the portable registration device. Still, it is hard to overestimate the significance of this paper as it proves the possibility of the development of a continuous cortisol sensor based on an antibody receptor.

Undoubtedly, great success has been achieved in measuring the concentration of cortisol on the surface of the skin in perspiration or in interstitial fluid in the subcutaneous layer using portable sensors [9,45,46]. Antibodies and aptamers were used as the primary receptors. The sensors consist of microfluidic chambers and microchips that allow the surface electrochemical phenomena or lateral transfer to be measured. Thus, in one of the earliest works, measurements were carried out in samples of interstitial fluid collected for 6 h through channels formed by a laser scalpel. The photolithographic technique was used to fabricate an oxidized silicon wafer in a clean room environment. Gold microelectrode arrays were functionalized with non-specified anti-cortisol (Mab) using a dithiobis (succinimidyl propionate) (DSP) self-assembled monolayer resulting in an ultrasensitive, disposable, electrochemical cortisol immunosensor using the electrochemical impedance (EIS) technique. This sensor was reusable, but not continuous and not reversible. However, it demonstrated the possibility of measuring the physiological concentrations of cortisol with a nM resolution using the EIS technique in combination with an antibody receptor immobilized on the gold microelectrodes. The sensitivity was further increased with functionalized antibodies specific to cortisol of the conductive carbon yarn (CCY) with ellipsoidal Fe_2_O_3_ [46]. Cortisol was measured in the collected sweat samples with cyclic voltammetry (CV), or a so-called electronic tongue, which is a powerful and popular electrochemical technique commonly employed to investigate the reduction and oxidation processes of molecular species [47]. A prototype of a wearable cortisol sensor based on a cortisol specific aptamer (a specifically designed oligonucleotide with high affinity binding to the ligand) and a platinum/graphene extended gate field effect transistor (EG-FET) as a detection method was recently described [48]. The measurements were conducted in the solution in vitro. However, the device shows promising parameters for the real-time monitoring of the cortisol in human sweat. Specifically, in the solution, hysteresis-free EG–FET curves were recorded from a 1 nM to 10 μM cortisol concentration with a detection limit of 0.2 nM with a negligible drift over time and high selectivity. However, low volume and a slow flow rate of the sweat might be an obstacle to the reversible continuous monitoring of cortisol concentration in vivo. Analytical performance, sample sources and analytical signal generation techniques of the recently developed immunosensors are summarized in Table 1.

Apparently, discussed analytical systems are performing with enough accuracy to measure cortisol concentrations fluctuations with antibodies as biorecognition element in vitro. Application of such assays to the novel surface substrates and development of adapted for in vivo signal acquisition strategies may result in CCM device prototype.

Some techniques discussed were developed using antibodies and aptamers (Table 2).

### 3.3. Molecularly Imprinted Polymer Based Cortisol Biorecognition

**Figure 8:**
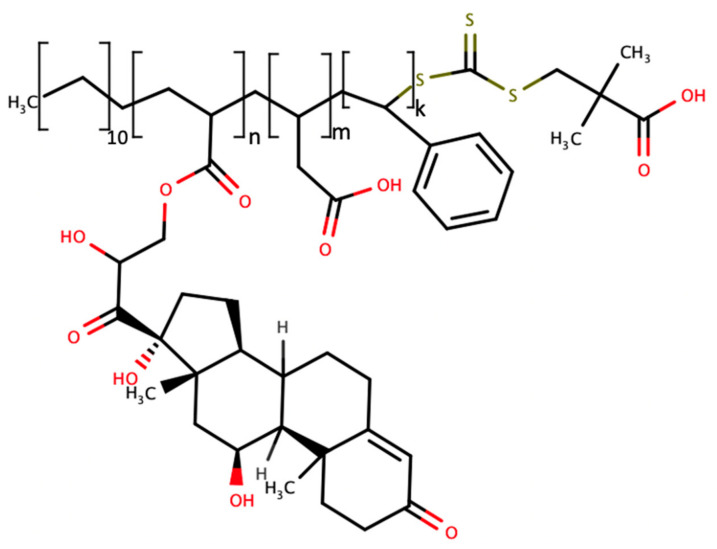
Polymer-bound cortisol hapten (acrylated cortisol) serves as a self-templating element that creates steroid-specific pockets on the surface of the SWCNT bound polymer loops formed by the introduction of hydrophobic copolymers anchoring to the SWCNT surface as well as flexible hydrophilic loops (n, m, k depicts the number of copolymer monomers in each functional segment of the polymer) ([59] Reprinted with permission from Wiley).

A biorecognition element formulated in the presence of the analyte—molecularly imprinted polymer (MIP)—is another example of nanochemistry used for the cortisol monitoring task. A self-imprinted polymer functionalized with the appendage steroid—acrylated cortisol—was created on the single-wall carbon nanotube (SWCNT) surface interpolymer pockets of the same size and surface charge as a free steroid. As the free steroid concentration increased, the acrylated appendage cortisol was easily displaced from the SWCNT surface, releasing the polymer loops farther from the SWCNT. Such flexible polymer loop movement changes the physico-chemical properties of the sensor to be detected as the analytical signal proportional to the free cortisol concentration (Figure 8) [59].

**Figure 9:**
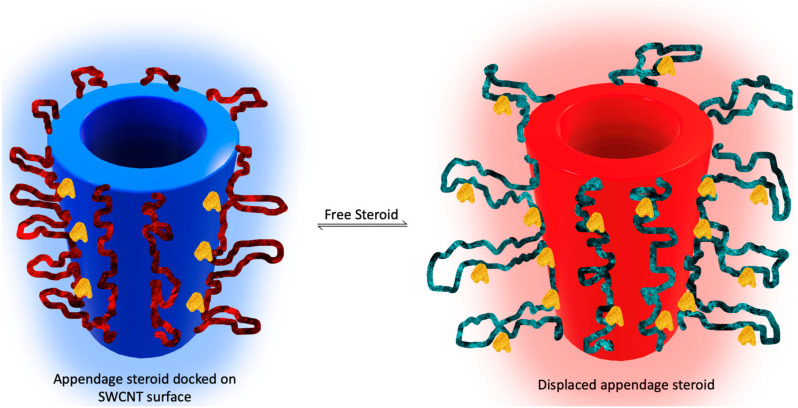
Simplified scheme for the illustration of the equilibrium reaction—displacement of the interpolymer-bound cortisol hapten (acrylated cortisol) by free cortisol molecules. Competing for docking closely at the SWCNT surface interpolymer pockets, free cortisol and hapten reconfiguring polymer loops change the nano-environment of the SWNT, producing the analytical signal based on the SWNT corona phase recognition measurement to monitor the free cortisol fluctuations over time in hydrogel, allowing for the free diffusion of steroids from the liquid samples in contact with them (Reproduced with permission from Wiley [59]).

However, these interpolymer pockets on the surface of single wall carbon nanotubes (Figure 9) appear to have less specificity to cortisol when performed with a mixture of steroids—cortisol and cortisone are measured by this system in an indistinguishable manner: the other natural steroids also show some reactivity toward self-imprinting with cortisol as the appendage polymer. Micromolar sensitivity is not sufficient for a normal nanomolar range of cortisol concentration in the serum, but the approach shows a dynamic response, allowing us to measure the cortisol concentration fluctuations over time, and could be further modified to produce a more specific response with aan optimized biorecognition element toward in vivo measurements.

Lee et al. demonstrated in vivo monitoring with such a SWCNT-doped hydrogel, but not for cortisol—several steroids were utilized to form a reactive looped imprinted polymer in a hydrogel, one of which, progesterone, was measured in vivo in mice by implanting a dialysis bag with an 8 kDa molecular cutoff to demonstrate the applicability of the CoPhMoRe sensor for the further development of continuous monitoring with such analytical principles.

Another example of the imprinted polymer for the cortisol monitoring task is the lossy-mode resonance measured from the partially decladded nanocomposite covered optical fiber [36]. The 600 µm core silica optical fiber was modified to create a sensing surface of 15 mm in length. The clad was removed in the waist-like shape of the sensing region in between the connectors to the light source from one side and the detector from another intact clad side of the probe. The composite was a 12 nm layer of lossy-mode resonance generating ZnO, covered with a molecularly imprinted polymer consisting of pockets of cortisol shape and charge compatible surface interfaces that are formed by template imprinting and removal prior measurements. The reported sensitivity in the range of 10^−12^–10^−6^ g/mL of cortisol by the reported technique meets the demands of the physiological cortisol concentration levels that need to be monitored in (artificial) saliva. However, the approach requires flow-through of the sample over the decladded sensing region in the optical fiber connected to the loop-like scheme of optical devices (light source, detector), making such a measurement setup not quite practical for continuous cortisol monitoring in vivo, but applicable as a low-volume test system. Molecularly imprinted polymers are often used as cortisol biorecognition element for cortisol sensing. Analytical performance of some of such assays is summarized in Table 3.

### 3.4. Alternative Protein Scaffolds for CCM

Alternative protein scaffolds exist and are applicable for cortisol sensing. Naturally, the glucocorticoid receptor (GR) ligand binding domain (LBD) is a biorecognition element. The affinity of the LBD of the mineralocorticoid receptor is 0.5 nM, which is typical for a high affinity cortisol receptor. The affinity of glucocorticoid receptor (GR) to cortisol was about 5 nM. The binding of cortisol to the LBD of GR results in GR conformational change, activating its biological activities in the downstream pathway. One of such activities of the ligand-bound GR is the binding of the transcriptional co-activators and co-repressors via specific amino acid motifs. One such co-activator, NCOR1, contains the LxLL motif, which is specifically bound by allosterically remodeled activated GR LBD reversely. This biological property of GR was utilized to develop GR LBD and NCOR1 chimeric proteins for intermolecular binding assays based on bio-luminescent probe function reconstitution [63,64,65]. As GR LBD binds free cortisol from the sample, it changes conformationally to obtain affinity to the LxLL motif [66], bending the chimeric protein in a way that two split luciferase domains are in close proximity, and regains its function by the substrate reaction emitting fluorescent light, with an intensity proportional to the cortisol concentration.

**Figure 10:**
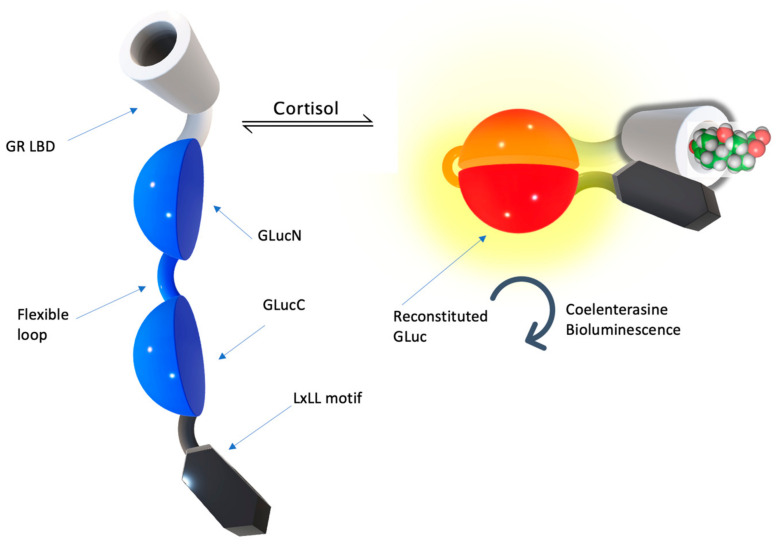
Intermolecular equilibrium binding of the split-luciferase cortisol fluorescent assay for in vitro cortisol monitoring. As cortisol appears and activates glucocorticoid LBD, which conformationally changes toward enhanced affinity to the LxLL motif, allowing two luciferase domains to reconstitute its function and to report a cortisol concentration increase (Reproduced from [65] with permission from @ACS).

Structural rearrangements in GR LBD change its biological properties, allowing the utilization of the switching affinity of GR to other components of transcription activation and the repression of this nuclear receptor was demonstrated to be useful in the development of a single-molecule fluorescent probe. This technique was developed by producing a chimeric protein of two split luciferase domains with a flexible linker in between. This complex is flanked with GR LBD from one side of the construction and its specific binding protein, NCOR1 (transcription activator), is the LxLL motif from the other side of the construction (Figure 10). As GR LBD binds free cortisol from the sample, it changes conformationally to increase the affinity to the LxLL motif, bending the chimeric protein in the way two split luciferase domains are in close proximity, and regains its function by the substrate reaction emitting fluorescent light, with an intensity proportional to the cortisol concentration.

Such proteins were expressed in vitro in the cells and immobilized in wells to function as a fluorescent test system to perform measurements of cortisol concentration in the saliva and serum, which demonstrated a high sensitivity and dynamic range, allowing us to measure the cortisol concentration in the volunteers’ saliva samples with its physiological concentrations of a 10^−8^ M range with corresponding results when compared with the ELISA measurement control method. Kim et al. [63,64,65] demonstrated the sensor performance across a wide range of concentrations, 10^−9^–10^−6^ M, effectively realizing a unimolecular biosensor with the intermolecular equilibrium binding signal generation principle. The authors stated that their cSimgr4 construction was selective only for cortisol and did not show any response on other steroids.

## 4. Discussion

The total cortisol levels in plasma varied from 80 to 700 nmol/L in normal subjects with 90% of cortisol in the plasma protein-bound. In saliva, all cortisol molecules are free and the concentration ranges from 1.5 to 15 nM [67]. To measure the differences in 1 nM increments of free cortisol, the affinity of the cortisol receptor in the sensor has to be at least in the nM range. This is comparable to the affinities of monoclonal antibodies to cortisol. The problem is that ligand binding to the receptor with Kd in nM can be considered as irreversible in the time intervals of 1 h, which is relevant to physiological measurements. Hence, the optimal balance between affinity and the half time of dissociation must be optimized for continuous measurements without chemical “recharging” of the receptor, as carried out in the salivary sensors by the repetitive acid washing of bound cortisol. To achieve continuous measurement, several individual recombinant antibodies to cortisol should be characterized, and the antibodies with optimal Kd based on reasonable sensitivity and dissociation time selected.Development of a clinically relevant (in terms of accuracy and rational sample source choice) continuous cortisol sensor has been stated as the ultimate goal by many research laboratories. However, the technical achievements of the numerous recent publications have not resolved all of the complex engineering tasks of an in vivo cortisol monitoring device. It includes an optimal time lag of the measurements due to the biorecognition reaction kinetics (up to 20 min [3]), allowing us to distinguish individual cortisol pulses, a proper protection of the sensing surface (from fouling and matrix background), the identification of the most relevant sample source, and the biocompatibility of the sensor components for long-term implantation of the sensing probe for in vivo measurements in the tissue of interest.

The effective membrane protection of such a prospective sensor could again be “borrowed” from the field of glucose sensing, where PSF hollow fiber membranes, designed for dialysis, are used to contain and protect electrode sensor. One should take into account the cortisol molecule’s physico-chemical properties, namely, the lipophilic steroid ability to aggregate and vessel wall stacking in polar solvents. Therefore, CCM device sensor membrane protection must be developed and optimized to balance the permeability of a fast rate of cortisol free diffusion but be selective enough to prevent the cortisol-converting enzymes and matrix from introducing errors into the measurement.

One more example of the cortisol monitoring capable assay publication points to one of the limitations of the redox and electrochemical biosensors, which is highly dependent on solution components such as potassium and sodium ions in biofluids, pH, other steroids, and charged molecules [38]. In order to protect the redox aptasensor, an oil layer has been introduced to fill the pores of a low-selective track membrane, and thin layers of different lipids have been studied to assess the cortisol free diffusion rate and sensor fouling over time [38]. The concept of protecting hydrophobic (lipophilic) analytes such as cortisol is supported by the results of the published paper of a 5 min response time on concentration fluctuation with a performance of over 7 h under oil membrane protection from pH 3 outside the sensor compartment.

It is also important to measure the free cortisol concentration inside the circulation in blood or within ISF, not in excreted fluids such as sweat and saliva. Indeed, only cortisol concentration in blood provides needed information on complex biological conditions [68,69].

While a substantial progress toward CCM device development has been documented in numerous publications, a time-resolved, biocompatible, biostable, compact, and effective biosensor to monitor cortisol rhythm accurately in vivo has not been described yet. A thorough description of specific stages available in recent publications allow us to predict some of the crucial features of future CCM analytical systems.

The probe of the sensor must be placed in interstitial fluid or into vascular space. The latter is more difficult to control and is hardly possible without a professional medical installation. Interstitial type sensors have been proven to be practical for continuous real-time glucose measurements. But, the Eversense capsule is still too large, a 18.3 mm of length and 3.5 mm in diameter, which is implanted with a special needle injector operated by trained medical personnel. A cortisol sensor will most likely utilize the design of a microneedle serving as a catheter to deliver a filament containing a modified optical fiber. Optical fibers are hair-thin and quite flexible and can be durable if it is of a polymer material. The rationale of measuring cortisol in the interstitial fluid is well-supported in the study by Venugopal et al. [53]. The electrochemical impedance (EIS) technique and ELISA have shown a high degree of correlation between ISF and the saliva concentrations of cortisol across diurnal variation.

It has been reported that approximately 70% higher cortisol numbers are seen in ISF. The authors concluded that ISF might be a better choice of a bodily fluid to be tested for levels of cortisol than saliva, serum, or blood. However, most of the published works on cortisol monitoring in ISF have been performed in vitro by external devices after the samples of ISF were collected and concentrated.

The interstitial fluid, unlike sweat or saliva, provides the dynamic milieu with constant flow. The concentration of cortisol in the interstitial fluid follows the fluctuation in the hormone concentration in the plasma. Cortisol is present in ISF mostly as a free hormone since the cortisol binding globulin (CBG) is cutoff by the endothelial layer of the vessels.

The receptor (antibody, aptamer or imprinted polymer) has to be optimized in terms of the thermodynamics of binding (affinity or dissociation constants) and the kinetics of association/dissociation. This will allow for both the detection of the hormone at a low concentration and with a sufficiently fast time resolution. An elegant theoretical study was recently published addressing this problem [69]. The study argued for a pre-equilibrium biosensor, in which the actual measurement reflects the receptor’s kinetic response and the algorithm quantifies the changing ligand concentration, analyzing the biosensor output in the frequency domain, rather than in the time domain. The authors concluded that antibodies or aptamers with a medium range (micromolar, rather than nanomolar affinity) should be used in such sensors. A different approach to the solution of the thermodynamics versus kinetics problem was taken by Lubken et al. [70].

It will be essential for a reversible sensor with high precision to use a single molecular interaction, as described in [43]. It was shown that a sensor based on an immobilized monoclonal antibody responded to cortisol in the high nanomolar to low micromolar range and could monitor cortisol concentrations over multiple hours. The measurement was made under a stationary microscope through the registration of the Brownian motion of the particles decorated with the antibody and tethered to the substrate by an extended DNA bridge. For a wearable sensor, this method must be modified, where the proximity of the reporter particle to the substrate will generate the optical signal.

Optical fiber-based sensing is a robustly developing area of research. Indeed, micro and nanofabrication, nanobiotechnological elements, and rationally exploited physico-chemical and optical properties of sensing waveguides are the variables for the construction of miniature, biocompatible, and sensitive analytical devices of great promise due to the utilization of fiber surfaces as assay substrates and signal transducing elements, which can be easily connected with existing light-guiding commutation hardware.

An optical fiber tip surface, modified with the capture antibody to adsorb S. aureus on its surface while a secondary antibody in complex with a quantum dot labelled reporter antibody could be detected by measuring the fluorescence on the fiber surface with a spectrometer attached to the probe through a bundle-waveguide. A 532 nm excitation laser light is delivered to the probe tip by the waveguide, and the backscattered QD emission from the bound S. aureus surface on the optical fiber tip is then read by the spectrometer attached to the bundle-waveguide [42].

**Figure 11:**
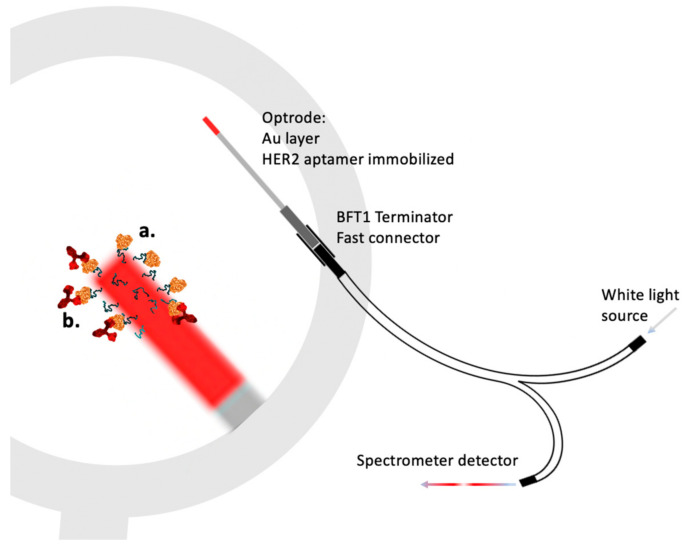
SPR optrode (a). First fast stage of HER2 capture by Au-bound aptamers. (b) Second long stage of the amplification of the signal with HER2 specific antibody adsorption on the fiber surface to enhance the signal 100-fold [71] with permission from Elsevier.

Another example of an optical fiber surface as a sensing probe and assay substrate is a multimode step-index silica core of 400 µm in diameter, silica in 25 µm TECS cladding, and a decladded 1-cm tip coated with 45 nm gold to obtain the surface-plasmon resonance (SPR) substrate. Such an optrode was further modified with aptamers catching HER2, a cancer biomarker that is the target for the bonding of the monoclonal antibody to HER2. The optrode is connected by the bundle-waveguide (Y-bundle, FC-PC, and BFT1 connectors of Thorlabs) to the white light source and the spectrometer reading the backscattered light (Figure 11) [71].

Resonance shift and refractive index changes over time were processed after calibration, which was performed with two modes of measurement: without amplification (only HER2 adsorption is detected, with fast reaction kinetics of around 10 min) and ~8 nM (1 µg/mL) sensitivity, and after ~40 min of incubation, the amplification of the signal reached 100-fold, as antibodies bound HER3 bound to the fiber surface by aptamers, enhancing the signal as the volume of the adsorbed complexes increased. Such amplification gains showed ~86 pm (10 ng/mL) of sensitivity [71]. Such examples of optic fiber probe development demonstrate the high practical value of side-emitting and collecting optical sensors for the development of prospective in vivo monitoring devices.

Taking into account the prospective CCM device analytical performance requirements and the key features of the considered analytical systems, here, a strategy is proposed to create a functional CCM prototype.

The metal-enhanced assay previously described in [39] could be applied to optical fiber sensing and optimized further by using the same approach of intermolecular binding simply to bend the chimeric protein probe to the surface of the nanostructured optical fiber probe sensing region. The minimal functional part of GR LBD could be fused through a flexible protein linker with the LxLL motif.

**Figure 12:**
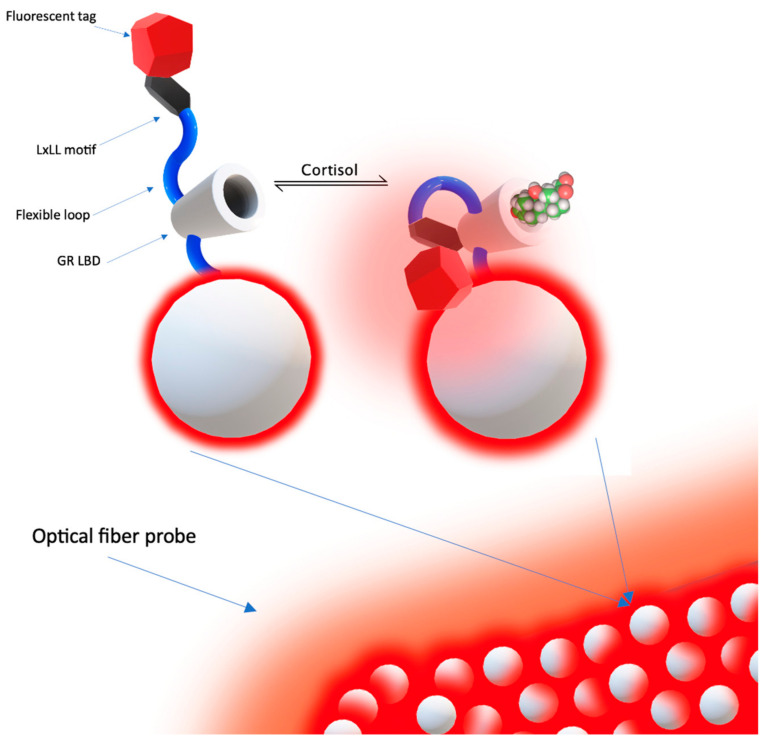
Intermolecular equilibrium binding of the proposed single-molecule metal-enhanced fluorescent assay on the polymer optical fiber toward the in vivo monitoring of cortisol.

The addition of a surface-specific tag will be used to immobilize the GR LBD to the AuNPs on the fiber surface. The sequential covalent attachment of a specific protein tag to the terminal of the chimeric protein oriented to the outward surface will result in bending the protein toward the nanostructured metal surface if the GR LBD is activated by free cortisol (Figure 12).

Binding of the protein probe will shorten the distance between the fluorescent reporter and Au nanoparticles on the surface of the fiber probe, which will increase the fluorescence intensity proportionally to the free cortisol concentration. Introducing such a multifunctional biorecognition element with a single cortisol binding site and fluorescent reporter tethered to the surface must be beneficial for the selectivity of the prominent sensor properties—the binding/dissociation kinetics, noise to signal ratio—and will probably decrease the technical demands for the protective membrane of the probe for in vivo measurements.

Such systems, based on the biosensing properties of the natural cortisol receptors together with a hair-thin, durable, flexible, and reliable surface substrate and signal transducing element, are promising strategies toward ideal dynamic biosensors for CCM in vivo.

## 5. Conclusions

A real-time continuous sensor for cortisol remains a desirable but still unreached goal, even regarding a laboratory prototype. However, there is an understanding of the main conditions that will allow for such a sensor to be built. All essential elements were already described and assessed experimentally. This includes a reversible receptor based on the monoclonal or engineered antibody and immobilized by a flexible bridge, allowing for a reversible single molecule interaction of the receptor with the analyte. The interaction of the fluorescent tag with the metal impregnated surface of the optical fiber provides a signal enhancement. We believe that “corticometers” applicable for laboratory research and clinical practice will be demonstrated soon, based on the principles outlined in our review.

## Figures and Tables

**Table 1:** Immunosensors.

Sample Source	Linear Range, LOD	Signal Generation	Reference	Applicability In Vivo
Saliva	0.001 ➔ to 10^4^ nM LOD (–)	Field effect transistorm (Liquid Gate, Graphene)	[49]	No
Urine and Serum	1 to 400 ng/mL 2.75 µM–1 mM LOD 3 ng/mL	Magnetically assisted SERS immunoassay (MA-SERSI)	[50]	No
Universal	Serum, blood 50–200 ng/mL (137 µM–0.5 mM) Saliva 1–40 ng/mL (2.75 µM–0.1 mM) Sweat 10–150 ng/mL (27 µM–0.4 µM) LOD 1 ng/mL	Non-faradaic electrochemical impedance spectroscopy (EIS)	[51]	No
Saliva	156~10,000 pg/mL (0.4–2.7 µM) LOD 10 pg/mL	Chemiresistor graphene oxide sensor	[52]	No
Dialyzed reconstituted Human plasma	100 nM–10 µM LOD (–)	Particle mobility assay	[43]	Yes; Pre-microdialysis required
Extracted ISF	1 pM to 100 nM LOD (–)	Electrochemical impedance immunoassay	[53]	Yes; ISF extractor required
Sweat	1 pg/mL to 150 ng/mL (2.75 nM–0.04 mM) LOD (–)	Chronoamperometry and non-faradaic electrochemical impedance spectroscopy (EIS)	[54]	No
Saliva	0.2–0.6 ng/mL LOD 0.005 ± 0.002 ng/mL	Field effect transistor	[55]	No
Buffer	2 to 50 ng/mL LOD 0.66 ng/mL	Electrochemical impedance immunoassay	[56]	No
Buffer	0.1 to 20 μg/mL LOD 0.02 μg/mL	Metal-enhanced fluoroimmunoassay	[39]	No
Reconstituted Human serum	5.0 10^−3^ and 150 ng mL^−1^ LOD 3.5 pg mL^−1^	Magnetic particle-assisted competitive immunoassay with differential pulse voltammetry	[57]	No
Preprocessed Saliva	0.1 ➔ µM to 10 mM LOD 100 pM	Quantum dots fluorescence quenching immunoassay	[40]	No

**Table 2:** Aptasensors.

Sample Source	Linear Range, LOD	Signal Generation	Reference	Applicability In Vivo
Sweat	1−256 ng/mL (2.75 µM–138 µM) LOD (–)	Electrochemical ZnO polymer matrix electrode	[6]	No
Saliva	50 to 200 nM LOD 10 pM	Metalloporphyrin based macrocyclic catalyst electrochemical sensor	[58]	No
Sweat	1 pM to 10 μM LOD 10 pM	Liquid-ion gated field-effect transistor (FET)	[48]	No

**Table 3:** MIP-based sensors.

Sample Source	Linear Range, LOD	Signal Generation	Reference	Applicability In Vivo
Artificial saliva	10^−12^ to 10^−6^ g/mL (2.75 pM to 2.75 µM) LOD 25.9 fg/mL	Fiber optic Lossy mode resonance ZnO/polypyrrole	[36]	No
Sweat	0.03 to 3.6 × 10^−6^ g/mL (9.9 µM–82 nM) LOD (–)	Electrochemical transistor	[60]	No
Sweat	10 ng/mL–60 ng/mL 27.5 µM–165 µMLOD 2.0 ng/mL ± 0.4 ng/mL	Electrochemical (PDMS doped with carbon nanotubes-cellulose crystals)	[61]	No
ISF	10 × 10^−6^ to 100 × 10^−6^ M (10–100 µM) LOD (–)	Corona phase molecular recognition (CoPhMoRe)	[59]	Yes (concept demonstrated on progesterone)
Saliva	0.5 nM to 64 nM LOD 0.14 nM	Impedimetric sensor	[62]	No

## Data Availability

Not applicable.
